# Does deterioration in mental health after smoking cessation predict relapse to smoking?

**DOI:** 10.1186/s12889-015-2473-z

**Published:** 2015-11-20

**Authors:** Gemma Taylor, Ann McNeill, Paul Aveyard

**Affiliations:** UK Centre for Tobacco and Alcohol Studies, School of Health and Population, Sciences, University of Birmingham, Birmingham, B15 2TT UK; UK Centre for Tobacco and Alcohol Studies, Institute of Psychiatry, King’s College London, London, SE5 8AF UK; UK Centre for Tobacco and Alcohol Studies, Nuffield Department of Primary Care Health Sciences, The University of Oxford, Oxford, OX1 2ET UK

**Keywords:** Smoking cessation, Tobacco, Epidemiology, Relapse, Mental health

## Abstract

**Background:**

It is possible that some people who quit smoking experience improved mental health after cessation and therefore remain abstinent, whereas other people who quit may experience worse mental health after cessation and therefore be more likely to relapse to smoking. Thus, in this study we aimed to examine the association between an enduring change in mental health following the cessation period and future risk of relapse.

**Methods:**

A secondary analysis of prospective data pooled from five placebo-controlled randomised trials for smoking reduction conducted in Europe, USA and Australia. Change in mental health (SF-36, scored 0–100) was measured from baseline to four months for those who were biologically-validated as point-prevalence abstainers at four month follow-up. Thereafter we assessed whether relapse to smoking by 12 months was more likely in those whose mental health had worsened between baseline and four months compared with those who saw no change or an improvement.

**Results:**

After adjustment for baseline mental health and other major covariates, there was no greater tendency to relapse at 12 months for those whose mental health worsened after cessation compared with those who had no change or an improvement. The odds ratio and 95 % confidence interval was 1.01 (0.97 to 1.05).

**Conclusions:**

People whose mental health worsens after smoking cessation are at no greater risk of subsequent relapse to smoking than those whose mental health stays the same or improves.

**Electronic supplementary material:**

The online version of this article (doi:10.1186/s12889-015-2473-z) contains supplementary material, which is available to authorized users.

## Background

A recent meta-analysis reported strong evidence of an association between stopping smoking and improved mental health [1]. This is surprising, because many smokers perceive that smoking benefits their mental health [2–7]. The misattribution hypothesis explains why smoking is perceived as offering mental health benefits but why stopping smoking will improve mental health [8–10]. Chronic tobacco smoking leads to periods of withdrawal which are characterized by restlessness, depressed mood, irritability and anxiety [11, 12]. The hypothesis recognises that these withdrawal symptoms are also a hallmark of many mental health disorders, but are reliably relieved by smoking despite only occurring because the person smokes in the first place. Thus the tobacco withdrawal cycle mimics symptoms of mental illness, while simultaneously misleading the smoker to believe that smoking offers psychological benefits. The model finally suggests that mental health will improve after cessation once the person who quit has surpassed the withdrawal period [11, 12] and tobacco-induced psychological withdrawal symptoms dissipate.

Although this provides a neat explanation for the findings, it is at odds with other data. Adolescents with mental health problems are more prone to take up smoking than healthy peers, adjusting for other possible causal factors [13, 14]. A Mendelian randomisation study suggests that smoking may not cause worse mental health [15]. Instead, the self-medication hypothesis proposes that smokers get genuine benefits from smoking and thus their mental health would deteriorate on cessation [16]. It is likely that these smokers may be at greater risk of returning to smoking or may even decide to return as their mental health is worse off without cigarettes than while smoking [17]. It is possible that different groups of smokers respond differently and hence both hypotheses might be true. This explanation implies that the finding that cessation improves mental health could apply to only some smokers. Other smokers might remain trapped in persistent smoking by deteriorating mental health when they try to stop. In this paper, we examine evidence for this hypothesis.

Previous studies have examined this association in the general population. Yong et al. [18] prospectively examined the association between emotional experiences after quitting and odds of relapsing versus remaining abstinent over a four year period. After cessation, participants rated questions such as, “Since you quit, has your ability to calm down when you feel stressed or upset improved, gotten worse or stayed the same?” People who perceived their ability to calm down had worsened were at greater risk of relapse to smoking by follow-up. These data were weakened because participants recalled a change, and this may have introduced bias [19–21]. It would have been preferable to assess change in mental health by measuring health on two occasions where people rated their current mental health with a validated tool. Gruder and colleagues [22] prospectively examined change in depression scores from baseline, when participants were smoking, to two year follow-up, when participants had either relapsed or quit. Results indicated that change in depression scores was not associated with relapse. Similarly, Manning et al. [23] analysed data from a cessation treatment trial to determine if change in stress from baseline (before quitting) to six month follow-up was associated with relapse at six month follow-up. Analyses indicated that there was no association between change in stress scores and relapse at follow-up. However, in these studies mental health was assessed after relapse had occurred, thus the data cannot assess whether deterioration in mental health after cessation predisposes to relapse.

Therefore, in this analysis we used data gathered prospectively in studies that recorded mental health at entry to a smoking reduction trial, and then for people who quit at four months after trial entry in the expectation that, for many, the acute withdrawal period would have passed. We then assessed whether the change in mental health between trial entry and four months was associated with subsequent relapse to smoking at 12 months.

## Methods

This study followed STROBE reporting guidelines for observational studies [24].

### Study design

This was a secondary analysis of prospective individual level patient data from five merged placebo-controlled randomised trials (RCTs) of nicotine replacement therapy (NRT) for smoking reduction which were conducted in Europe, USA and Australia. These data were provided by McNeil pharmaceutical company, who played no role in analysis, data interpretation or the writing of this paper. All these trials were carried out to a consistent protocol which included adults who smoked for at least three years and were motivated to reduce their smoking, but not to quit during the first month of the trial. Smoking cessation was recommended as the ultimate goal throughout the trials, but was not mandatory. Participants were excluded if they were deemed medically unfit by a practitioner, pregnant or breastfeeding or partaking in another smoking intervention (see reports of trials for further details [25–28]. These trials measured the variables of interest to this study consistently at four and 12 month follow-ups, therefore to maximise the sample size we chose these time points.

### Ethics statement

Each trial received ethical approval from the appropriate bodies within the country it was conducted, and trials were conducted in accordance with the ethical principles outlined in the Declaration of Helsinki. See reports of trials for further details [25–28].

### Consent statement

All participants provided informed consent to participate in the trials. See reports of trials for further details [25–28].

### Participants and study size

Seven-hundred-and-forty-one participants enrolled in the trials provided data at baseline, four and 12 month follow-up. One hundred and seven participants were biologically-validated as quit at four month follow-up. Of these, 80 participants provided sufficient data at 12 month follow-up to determine their relapse to smoking status with biological validation and this abstinent group formed the cohort for this study.

### Variables

#### Exposure

The exposure was defined as change in mental health from when the person was a smoker at trial entry (baseline), to after they had been abstinent for at least seven days at the four month follow up. All cessation outcomes were biologically verified by exhaled carbon monoxide (CO < 10 ppm). Mental health was measured using the “emotional wellbeing” subscale from the RAND-36/SF-36 item health survey 1.0. Scores range from 0 to 100, an increase on the scale indicates improved mental health and scores of ≤38 indicates presence of a mental health problem. In the general population the subscale mean and standard deviation are 70 [22, 29, 30]. The scale correlates highly with other mental health measures, and is available in multiple languages and valid in many countries [31].

### Outcome

**Fig. 1:**
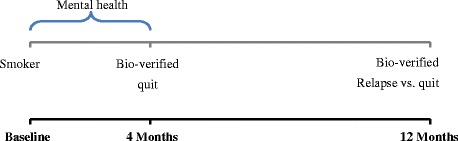
Timeframe for measurement of exposure and outcome variables

The outcome was bio-validated smoking at 12 months or bio-validated point-prevalence abstinence at 12 months. Those whose carbon monoxide level did not confirm either smoking or quitting at four or 12 month follow-ups were not included in the analysis. Figure 1 explains the measurement of exposure and outcome.

### Confounding variables

The following variables were potential confounders of the association between change in mental health and quit attempt success: Nicotine dependence, as measured using the Fagerström Test of Nicotine Dependence (FTND) [32]. Higher nicotine dependence has been found to be associated with worse mental health and quit attempt success [3, 34]. NRT treatment status (placebo or active) was added as active treatment has been found associated with quit success [35]. Sex and age have both been found associated with smoking status and mental health [36]. Baseline mental health scores (SF-36) may predict quit success and will also be the strongest predictors of inter-individual change [37–39].

### Statistical methods

To maximise the power of the analyses, we examined the association between change in mental health between baseline and four months and risk of relapse as a linear term, rather than dichotomising the data [40]. Logistic regression modelling was used to assess the association between change in mental health and relapse, a dummy variable was created for smoking status, repeated-point prevalence abstinence was indicated by ‘0’, and relapse indicated by ‘1’. We inversely scaled change in mental health by subtracting four month follow-up scores from baseline scores. The odds ratios therefore represented the odds of relapsing for a deterioration in mental health. The regression model was repeated with and without adjustment for FTND score, NRT treatment status, baseline mental health (SF-36), trial, sex and age.

### Sensitivity analysis

**Fig. 2:**
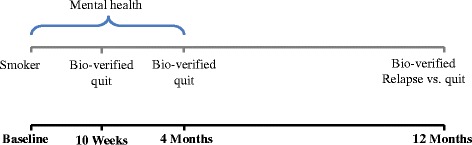
Timeframe for measurement of exposure and outcome variables in the sensitivity analysis

We chose the four-month follow up point so as to maximise opportunity to avoid the acute withdrawal period after cessation, but, given the quit date varied for smokers who stopped, the analysis could have included only recently abstinent smokers whose mental health could have deteriorated because they were suffering withdrawal. We were interested in those smokers who had been through the acute withdrawal period and subsequently suffered a more enduring deterioration in mental health. We addressed this in a sensitivity analysis by repeating the analysis excluding recently abstinent smokers and included only those abstinent at both 10 week and 4 month follow-up (Fig. 2).

### Risk of bias

An adapted version of the Newcastle-Ottawa Scale was used to assess risk of bias in observational studies [1, 41]. The measure rates studies on a scale of 1 to 5: 1 indicates a high risk of bias and 5 indicates a low risk of bias.

## Results

### Participants

One-hundred-and-seven participants were biologically-validated as quit at four months. Of these, 80 reported smoking status at 12 months. The 27 participants who did not provide smoking data at 12 months and who were excluded from the analysis were on average psychologically healthy, according to the SF-36 [29], with a mean (M) and standard deviation (SD) of 71.7 (21.2). Of the 80 participants reporting outcome data at four and 12 months, two people who quit were excluded as they did not have data on mental health. After excluding for missing data, the analysis included 17 people who quit (21 %) classified as relapsed to smoking at 12 month follow-up, and 61 as quit at 12 month follow-up.

### Characteristics of people who quit and those who relapsed to smoking

Baseline characteristics of people who quit and those who relapsed are presented in Table 1. The groups were not significantly different in age, sex, nicotine dependency (FTND), baseline mental health (SF-36) or receipt of active NRT (Table 1).

**Table 1:** Baseline (trial entry) characteristics of people who were quit or relapsed at 12 month follow-ups

Characteristic	Quit at 12 months (*N* = 61)	Relapsed at 12 months (*N* = 17)	*P*-value
Age, M (SD)	46.9 (9.6)	45.3 (11.9)	0.58
Sex, % male (*N*)	49 % (30)	71 % (12)	0.12
FTND, M (SD)	5.5 (2.4)	5.6 (1.5)	0.80
SF-36 Mental health, M (SD)	74.5 (14.7)	69.6 (18.0)	0.24
Treatment status, % received active (*N*)	72 % (44)	71 % (12)	0.90

### Logistic regression analyses

On average, both those who reported smoking relapse and those who remained abstinent reported small and similar improvements in mental health between baseline (smoking) and four month follow-up (abstinent). The mean (SD) improvements were 2.2 (14.8), and 3.1 (17.6) respectively. Mental health deteriorated between baseline and four months in 50 % (*n* = 30) of people who would remain abstinent to 12 months and deteriorated in 55 % (*n* = 6) of people who would relapse by 12 months.

The unadjusted model indicated that change in mental health from baseline to four months was not associated with odds of relapsing, compared with staying quit, odds ratio and 95 % confidence intervals (OR; 95 % CI) was 1.00 (0.96 to 1.03), *P* = 0.84. After adjustment for baseline mental health, FTND scores, NRT status, trial, age and sex, the association remained non-significant, 1.01 (0.97 to 1.05), *P* = 0.65 (Table 2).

**Table 2:** SF-36 Mental health scores at four and 12 month follow-ups for people who quit or relapsed by 12 month

	Quit at 12 months (*N* = 61)	Relapsed at 12 months (*N* = 17)
SF-36 Mental health at 4 month follow-up, M (SD)^a^	76.7 (13.7)	72.7 (19.5)
SF-36 Mental health at 12 month follow-up, M (SD)^a^	77.1 (15.8)	70.4 (16.3)

^a^No significant differences between groups SF-36 scores at 4 or 12 month follow-ups. Mean scores indicated the groups were psychologically healthy at both follow-ups. ^28^

### Sensitivity analysis

Excluding people who quit at four months (*N* = 28) did not change the results. Of the five people who relapsed, and the 45 people who were quit (repeated point-prevalence), there remained no association between change in mental health from baseline to four month follow, and relapse at 12 month follow-up, OR (95 % CI) was 1.01 (0.95 to 1.07), *P* = 0.73. Adjustment for covariates did not change the association, OR 1.01 (0.94 to 1.08), *P* = 0.82.

### Risk of bias

The study scored 4 out of 5, indicating a low risk of bias [41], one point was lost for high attrition (Additional file 1).

## Discussion

In this study we aimed to examine whether an enduring deterioration in mental health following smoking cessation was associated with future relapse to smoking. On average, people who quit and those who relapsed to smoking showed small improvements in mental health after they stopped smoking. However, some showed a deterioration but there was no evidence that worsening mental health between baseline and four months was associated with relapse to smoking by 12 months.

### Limitations, strengths and potential sources of bias

Although the cohort was small, the confidence intervals were narrow enough to exclude the possibility of even a moderate sized association. The use of a validated tool to measure mental health assessed on two occasions to assess change in mental health added validity [29]. In this dataset we were able to assess, for a subsample, mental health after the acute withdrawal period following cessation, thereby measuring a more enduring change in mental health. It is possible however, that some people whose mental health deteriorated over and beyond the acute withdrawal stage had already relapsed by four months and hence were not captured in our cohort. In addition, the trials analysed in this study excluded participants if they were under the care of a psychiatrist, thus these findings may not be generalizable to people with a longstanding or severe mental illness.

Pre-cessation scores were measured at baseline while everyone was a smoker and had no immediate plans to quit, only to reduce. It is possible that smokers who were motivated to reduce had better mental health than those who were not motivated, and this may limit generalisability of the results; however, there are no available studies to support this possibility. Post-cessation mental health scores were obtained at four month follow-up, when smokers had stopped and were proven to have stopped. Use of point-prevalence criteria to ascertain abstinence may have included some people who were not continuously abstinent for at least 6 weeks at the 4 month follow-up [42, 43]; thus some participants may have been experiencing withdrawal, in-turn influencing mental health scores [11, 12]. However a sensitivity analysis confined to people who were continuously abstinent at both 10 week and four month follow-up gave very similar results, therefore it is unlikely that mental health scores were affected. We used biologically-validated point-prevalence abstinence to define relapse between four and 12 months. It is possible that those who were biologically-validated as quit at both time-points may have relapsed but resumed abstinence and this possible outcome misclassification may underestimate the true strength of the association. However, it would be uncommon to find a smoker who was quit at both time points but had smoked in between [42, 43] and therefore we believe the degree of underestimation to be minimal.

### Interpretation

The finding of this study concurs with other studies that assessed mental health with psychometrically appropriate measures [22, 23]. Gruder et al. found that change in symptoms of depression and stress from baseline to follow-up were not associated with relapse to smoking [22], and Manning and colleagues [23] replicated these findings for symptoms of stress. However, these studies assessed mental health after relapse, meaning that it is possible that relapsing and reinstatement of the therapeutic benefits of smoking masked the association between worsening mental health and relapse. We did not have the same difficulty in this study. In addition, these other studies had odds ratios for the association close to one which were not changed much by adjustment, as in our study, but our study provides greater precision. The only data contradicting this study were reported by Yong et al. [18], which showed that smokers who reported that their ability to cope with negative affect had deteriorated after abstinence were more likely to relapse in the future. However, their study did not assess mental health when smoking and when abstinent. Reports of mental health change were based upon participants’ memory and this may have introduced bias. Reports of emotional symptoms from memory are well known to become less accurate and more biased over time; [19, 44–48] making the validity of such reports suspect and clouding the reported association. Given we found no evidence of this in a more robust design, we suggest the evidence does not favour this hypothesis.

## Conclusions

We found no evidence that there is an increased risk of relapse to smoking for those whose mental health worsens. It may be that the apparent improvement in mental health on cessation is likely to apply to all smokers. These findings have implications for cessation treatment in allaying concerns that smokers will not be able to emotionally cope without cigarettes and thus leading them to return to smoking. The findings of Yong and colleagues could suggest that smokers who believe that smoking is stress relieving are at increased risk of relapse [18]. It is now possible to address this belief directly. There is clear evidence from a systematic review and meta-analysis that mental health improves in most people who stop smoking and that it does not worsen in general [1], and, even for those that experience a worsening, this may not cause relapse. In addition to this reassurance, providing alternative and well-practised methods to manage stress may prevent relapse caused by this belief.

## Additional file

Additional file 1: **Supplementary material.** (DOCX 18 kb)

## Abbreviations

FTND: Fagerström test of nicotine dependence
NRT: Nicotine replacement therapy
RCT: Randomised controlled trial
SF-36 (RAND-36): Short-Form health survey
STROBE: Strengthening The Reporting Of Observational Studies In Epidemiology

## References

[1] Taylor G, McNeill A, Girling A, Farley A, Lindson N, Aveyard P (2014). Change in mental health after smoking cessation: systematic review and meta-analysis. BMJ.

[2] Fidler J, West R (2009). Self-perceived smoking motives and their correlates in a general population sample. Nicotine Tob Res.

[3] Kerr S, Watson H, Tolson D, Lough M, Brown M (2006). Smoking after the age of 65 years: a qualitative exploration of older current and former smokers' views on smoking, stopping smoking, and smoking cessation resources and services. Health Soc Care Community.

[4] Twyman L, Bonevski B, Paul C, Bryant J (2014). Perceived barriers to smoking cessation in selected vulnerable groups: a systematic review of the qualitative and quantitative literature. BMJ Open.

[5] Lawn SJ, Pols RG, Barber JG (2002). Smoking and quitting: a qualitative study with community-living psychiatric clients. Soc Sci Med.

[6] McEwen A, West R, McRobbie H (2008). Motives for smoking and their correlates in clients attending Stop Smoking treatment services. Nicotine Tob Res.

[7] Thompson B, Thompson L, Thompson J, Fredickson C, Bishop S (2003). Heavy smokers: a qualitative analysis of attitudes and beliefs concerning cessation and continued smoking. Nicotine Tob Res.

[8] Parrott A (1995). Smoking cessation leads to reduced stress, but why?. Subst Use Misuse.

[9] Parrott A (1998). Nesbitt's paradox resolved? Stress and arousal modulation during cigarette smoking. Addict.

[10] Parrott A (1999). Does cigarette smoking cause stress?. Am Psychol.

[11] Hughes J (2007). Effects of abstinence from tobacco: valid symptoms and time course. Nicotine Tob Res.

[12] Hughes J (2007). Measurement of the effects of abstinence from tobacco: a qualitative review. Psychol Addict Behav.

[13] Jamal M, Does A, Penninx B, Cuijpers P (2011). Age at smoking onset and the onset of depression and anxiety disorders. Nicotine Tob Res.

[14] Johnson J, Cohen P, Pine D, Klein D, Kasen S, Brook J (2000). Association between cigarette smoking and anxiety disorders during adolescence and early adulthood. JAMA.

[15] Bjorngaard J, Gunnell D, Elvestad M, Smith D, Skorpen F, Krokan H (2013). The causal role of smoking in anxiety and depression: a Mendelian randomization analysis of the HUNT study. Psychol Med.

[16] Khantzian E (1997). The self-medication hypothesis of substance use disorders: a reconsideration and recent applications. Harv Rev Psychiatry.

[17] Hughes J (2007). Depression during tobacco abstinence. Nicotine Tob Res.

[18] Yong HH, Borland R, Cooper J, Cummings KM (2010). Postquitting experiences and expectations of adult smokers and their association with subsequent relapse: Findings from the International Tobacco Control (ITC) Four Country Survey. Nicotine Tob Res.

[19] Coughlin S (1990). Recall bias in epidemiologic studies. J Clin Epidemiol.

[20] Hassan E (2006). Recall bias can be a threat to retrospective and prospective research designs. Internet J Epidemiol.

[21] Wood J, Garb H, Nezworski T. Psychometrics: Better measurement makes better clinicians. In: Lilienfeld S, Donohue W, editors. The Great Ideas of Clinical Science: 17 Principles that Every Mental Health Professional Should Understand. London, UK: Taylor & Francis Group; 2007.

[22] Gruder C, Trinidad D, Palmer P, Xie B, Li L, Johnson C (2013). Tobacco smoking, quitting, and relapsing among adult males in Mainland China: the China Seven Cities Study. Nicotine Tob Res.

[23] Manning B, Catley D, Harris K, Mayo M, Ahluwalia J (2005). Stress and quitting among African American smokers. J Behav Med.

[24] Von Elm E, Altman D, Egger M, Pocock S, Gøtzsche P, Vandenbroucke J (2007). The Strengthening the Reporting of Observational Studies in Epidemiology (STROBE) statement: guidelines for reporting observational studies. Prev Med.

[25] Batra A, Klingler K, Landfeldt B, Friederich H, Westin À, Danielsson T (2005). Smoking reduction treatment with 4-mg nicotine gum: a double-blind, randomized, placebo-controlled study. Clin Pharmacol Ther.

[26] Bolliger C, Zellweger J, Danielsson T, van Biljon X, Robidou A, Westin À (2000). Smoking reduction with oral nicotine inhalers: double blind, randomised clinical trial of efficacy and safety. BMJ.

[27] Rennard S, Glover E, Leischow S, Daughton D, Glover P, Muramoto M (2006). Efficacy of the nicotine inhaler in smoking reduction: a double-blind, randomized trial. Nicotine Tob Res.

[28] Wennike P, Danielsson T, Landfeldt B, Westin À, Tonnesen P (2003). Smoking reduction promotes smoking cessation: results from a double blind, randomized, placebo-controlled trial of nicotine gum with 2-year follow-up. Addict.

[29] Hays R, Prince-Embury S, Chen H (1998). RAND-36 Health Status Inventory.

[30] Hays R, Sherbourne C, Mazel R (1993). The RAND 36-item Health Survey 1.0. Health Econ.

[31] Hays R, Morales L (2001). The RAND-36 measure of health-related quality of life. Ann Med.

[32] Fagerström K, Heatherton T, Kozlowski L (1990). Nicotine addiction and its assessment. Ear Nose Throat J.

[33] John U, Meyer C, Rumpf H, Hapke U (2004). Smoking, nicotine dependence and psychiatric comorbidity - a population-based study including smoking cessation after three years. Drug Alcohol Depend.

[34] Vangeli E, Stapleton J, Smit E, Borland R, West R (2011). Predictors of attempts to stop smoking and their success in adult general population samples: a systematic review. Addict.

[35] Stead L, Perera R, Bullen C, Mant D, Hartmann-Boyce J, Cahill K, et al. Nicotine replacement therapy for smoking cessation. Cochrane Database Syst Rev. 2012;1. 10.1002/14651858.CD000146.pub4 23152200

[36] Van Loon A, Tijhuis M, Surtees P, Ormel J (2005). Determinants of smoking status: cross-sectional data on smoking initiation and cessation. Eur J Public Health.

[37] Asendorpf J (1992). Beyond stability: predicting inter−individual differences in intra−individual change. Euro J Personality.

[38] Barnett A, van der Pols J, Dobson A (2005). Regression to the mean: what it is and how to deal with it. Int J Epidemiol.

[39] Niaura R, Britt D, Shadel W, Goldstein M, Abrams D, Brown R (2001). Symptoms of depression and survival experience among three samples of smokers trying to quit. Psychol Addict Behav.

[40] McCaffrey D, Elliott M (2008). Power of tests for a dichotomous independent variable measured with error. Health Serv Res.

[41] Wells G, Shea B, O'Connell D, Peterson J, Welch V, Losos M, et al. The Newcastle-Ottawa Scale (NOS) for assessing the quality of nonrandomised studies in meta-analyses. Ottawa: 2010 2010. Report No.

[42] Hughes J, Carpenter M, Naud S (2010). Do point prevalence and prolonged abstinence measures produce similar results in smoking cessation studies? A systematic review. Nicotine Tob Res.

[43] Hughes J, Keely J, Niaura R, Ossip-Klein D, Richmond R, Swan G (2003). Measures of abstinence in clinical trials: issues and recommendations. Nicotine Tob Res.

[44] Henkel L, Mather M (2007). Memory attributions for choices: how beliefs shape our memories. J Mem Lang.

[45] Koriat A, Goldsmith M, Pansky A (2000). Toward a psychology of memory accuracy. Annu Rev Psychol.

[46] Robinson M, Clore G (2002). Belief and feeling: evidence for an accessibility model of emotional self-report. Psychol Bull.

[47] Robinson M, Clore G (2002). Episodic and semantic knowledge in emotional self-report: evidence for two judgment processes. J Pers Soc Psychol.

[48] Shiffman S, Hufford M, Hickcox M, Paty J, Gnys M, Kassel J (1997). Remember that? A comparison of real-time versus retrospective recall of smoking lapses. J Consult Clin Psychol.

