# Supplementary material for: Does deterioration in mental health after smoking cessation predict relapse to smoking?
Source: BMC Public Health. 2015 Nov 20;15:1150. doi: 10.1186/s12889-015-2473-z (PMC4654809; doi:10.1186/s12889-015-2473-z)
Supplement: Supplementary file 1 — Supplementary material. (DOCX 18 kb) [file 12889_2015_2473_MOESM1_ESM.docx]

**SUPPLEMENTARY MATERIAL**

| **Appendix 1: Quality assessment of study** | | | | |
| --- | --- | --- | --- | --- |
| **Newcastle-Ottawa quality assessment scale cohort studies (NOS) adapted version (Taylor et al, 2014)** | | | | |
|  | | **Star awarded system** | **Star (*) awarded** | **GT reasons** |
| **Study’s selection criteria** | | | |  |
| 1) Representativeness of the exposed cohort (maximum 1 star) | a) truly representative of the average ______ (describe) in the community | * |  |  |
|  | b) somewhat representative of the average _______ in the community | * | * | Not motivated to quit, they were motivated to reduce. |
|  | c) selected group of users e.g.. nurses, volunteers | (no star) |  |  |
|  | d) no description of the derivation of the cohort | (no star) |  |  |
| 2) Selection of the non-exposed cohort (maximum 1 star) | a) drawn from the same community as the exposed cohort | * | * | Both groups were derived from the same sample. |
|  | b) drawn from a different source | (no star) |  |  |
|  | c) no description of the derivation of the non-exposed cohort | (no star) |  |  |
| 1) Assessment of exposure (maximum 1 star)1 | a) standardised self-report questionnaire | * | * | Sf-36 |
|  | b) standardised interview schedule with blind assessor | * |  |  |
|  | c) non-standardised self-report questionnaire or non-standardised interview schedule | (no star) |  |  |
|  | d) no description | (no star) |  |  |
| **Study’s outcome criteria** | | | |  |
| Assessment of outcome (maximum 1 star) | a) Biologically-validated smoking status | * | * | Biologically-validated relapse status from 4 to 12 months |
|  | b) smoking status validated only by self-report | (no star) |  |  |
|  | c) no description | (no star) |  |  |
| 2) Adequacy of follow-up of cohorts (maximum 1 star) 2 | a) complete follow-up - all subjects accounted for  | * |  |  |
|  | b) subjects lost to follow-up unlikely to introduce bias - small number lost <___ % (select an adequate %) follow-up, or description provided of those lost)  | * |  |  |
|  | c) follow-up rate < __% (select an adequate %) and no description of those lost | (no star) |  | Overall 47% loss to follow-up. |
|  | d) no statement | (no star) |  |  |
| Final Score | | 4* | | |
| 1 In the case that a study has used a standardised self-report questionnaire and a standardised interview schedule, only one star will be awarded. This is as use of both outcome assessments will not improve the study’s quality for the purpose of this review.  2 Describe based on attrition from enrolment to final follow-up | | | | |
